# Abuse of Prescription Drugs in the Context of Novel Psychoactive Substances (NPS): A Systematic Review

**DOI:** 10.3390/brainsci8040073

**Published:** 2018-04-22

**Authors:** Fabrizio Schifano, Stefania Chiappini, John M. Corkery, Amira Guirguis

**Affiliations:** Psychopharmacology, Drug Misuse and Novel Psychoactive Substances Research Unit, School of Life and Medical Sciences, University of Hertfordshire, Hertfordshire AL10 9AB, UK; f.schifano@herts.ac.uk (F.S.); j.corkery@herts.ac.uk (J.M.C.); a.guirguis2@herts.ac.uk (A.G.)

**Keywords:** drug abuse, novel psychoactive substances, NPS, pharmacovigilance, prescribing drugs’ abuse

## Abstract

Recently, a range of prescription and over-the-counter drugs have been reportedly used as Novel Psychoactive Substances (NPS), due to their potential for abuse resulting from their high dosage/idiosyncratic methods of self-administration. This paper provides a systematic review of the topic, focusing on a range of medications which have emerged as being used recreationally, either on their own or in combination with NPS. Among gabapentinoids, pregabalin may present with higher addictive liability levels than gabapentin, with pregabalin being mostly identified in the context of opioid, polydrug intake. For antidepressants, their dopaminergic, stimulant-like, bupropion activities may explain their recreational value and diversion from the therapeutic intended use. In some vulnerable clients, a high dosage of venlafaxine (‘baby ecstasy’) is ingested for recreational purposes, whilst the occurrence of a clinically-relevant withdrawal syndrome may be a significant issue for all venlafaxine-treated patients. Considering second generation antipsychotics, olanzapine appears to be ingested at very large dosages as an ‘ideal trip terminator’, whilst the immediate-release quetiapine formulation may possess proper abuse liability levels. Within the image- and performance- enhancing drugs (IPEDs) group, the beta-2 agonist clenbuterol (‘size zero pill’) is reported to be self-administered for aggressive slimming purposes. Finally, high/very high dosage ingestion of the antidiarrhoeal loperamide has shown recent increasing levels of popularity due to its central recreational, anti-withdrawal, opiatergic effects. The emerging abuse of prescription drugs within the context of a rapidly modifying drug scenario represents a challenge for psychiatry, public health and drug-control policies.

## 1. Introduction

Novel Psychoactive Substances (NPS; ‘legal highs’ or ‘research chemicals’) are molecules designed to mimic the effects of legal traditional recreational drugs with intense psychoactive effects and virtual non-detectability in routine drug screenings. NPS include synthetic cannabinoids, cathinone derivatives, psychedelic phenethylamines, novel stimulants, synthetic opioids, tryptamine derivatives, phencyclidine-like dissociatives, piperazines, psychoactive plants/herbs and a range of prescribed medications [1]. The term NPS was first used by United Nations Office on Drugs and Crime (UNODC) to refer to “substances of abuse, either in a pure form or a preparation, that are not controlled by the 1961 Single Convention on Narcotic Drugs or the 1961 Convention on Psychotropic Substances, but which may pose a public health threat” [2]. At present, the emergence of NPS, typically from outside Western countries [3], represents a considerable public health challenge. Moreover, in order to circumvent the present controls and regulations, NPS are constantly diversifying and being replaced [4]. This is being facilitated by the growing number of anonymous online marketplaces, called ‘cryptomarkets’, which host many anonymous sellers whilst using untraceable cryptocurrencies [5]. NPS users report a range of reasons behind their preference for NPS as opposed to traditional drugs such as cannabis, cocaine and heroin, including typical lack of detectability, greater affordability, lack of stigma, and relative ease of online acquisition [6]. Recently, however, the phenomenon of using prescription drugs in an idiosyncratic way to resemble, or counteract, the effects of NPS, has increasingly been described. This phenomenon refers not only to high potency opioids (e.g., fentanyl) and ‘exotic’/designer benzodiazepines—molecules already having been reported to be addictive [1]—but also: gabapentinoids [7], a range of stimulants [1], antipsychotics [8], antidepressants [9] and image- and performance-enhancing drugs (IPEDS, e.g., anabolic steroids, vitamins, clenbuterol and salbutamol) [10]. Among over-the-counter drugs, the two most common agents reportedly ingested in intentional abuse cases are the antitussive, dextromethorphan [11], and loperamide, a common antidiarrhoeal drug [12].

Any pharmacovigilance approach aims to detect, assess, understand and hopefully prevent adverse effects or any other medicine-related problems. From this point of view, there is a growing attention on prescription drugs and their addictive liability levels/diversion potential [7,8,10,12]. As the intended and the actual use of medicines differ between clinical trials and real-world use, pharmacovigilance activities are well placed to focus on the post-marketing phase. In Europe, those activities are coordinated by the European Medicines Agency (EMA) [13] through EudraVigilance (EV), which is the system for collecting, managing and analyzing information on suspected adverse reactions to medicines which have been authorized in the European Economic Area (EEA) [14].

This paper aims to provide a systematic review of the available literature relating to a preselected range of prescription medicines (pregabalin, gabapentin, quetiapine, olanzapine, venlafaxine, bupropion, loperamide, clenbuterol and salbutamol) previously reported as possibly being misused as NPS. For each molecule, a range of preclinical, epidemiological, and clinical pharmacological data will be provided.

## 2. Materials and Methods

A systematic review was carried out, consistent with the Preferred Reporting Items for Systematic Reviews and Meta-Analysis (PRISMA) guidelines [15]. A literature search was performed on PubMed, Medline/OvidSP (includes Embase), and Web-of-Science; the current search was completed in February 2018 and was not associated with any time restrictions. We focused on pregabalin, gabapentin, quetiapine, olanzapine, venlafaxine, bupropion, clenbuterol, salbutamol and loperamide [Title/Abstract]. For each molecule, a number of search terms [Title/Abstract] were considered as follows: ‘misuse’, ‘abuse’, ‘dependence’, ‘withdrawal’, ‘off-label use’ and ‘non-medical use’. In addition, the authors performed further secondary searches by using the reference listing of all eligible papers. All titles/abstracts were examined, and full texts of potentially relevant papers obtained. Relevant works were selected in order to obtain a full representation of the available literature data on the selected topic. Eligible studies were identified if they possessed a range of characteristics, including (1) peer-reviewed clinical/human studies; (2) at least an abstract with estimates and/or full availability of results; and (3) focusing on the misuse/abuse/dependence/withdrawal of pregabalin; gabapentin; quetiapine; olanzapine, venlafaxine, bupropion, loperamide, clenbuterol and salbutamol. The entire range of literature papers were included, e.g., experimental and observational studies; case reports; case series; and fatalities’ reports. Although letters to the editor, conference proceedings, and book chapters were excluded from the systematic review, they were still considered in the retrieval of further secondary searches. SC independently extracted and collected relevant data; FS contributed to the analysis of the results and discussed possible issues and disagreements during the revision of the paper with SC.

**Figure 1:**
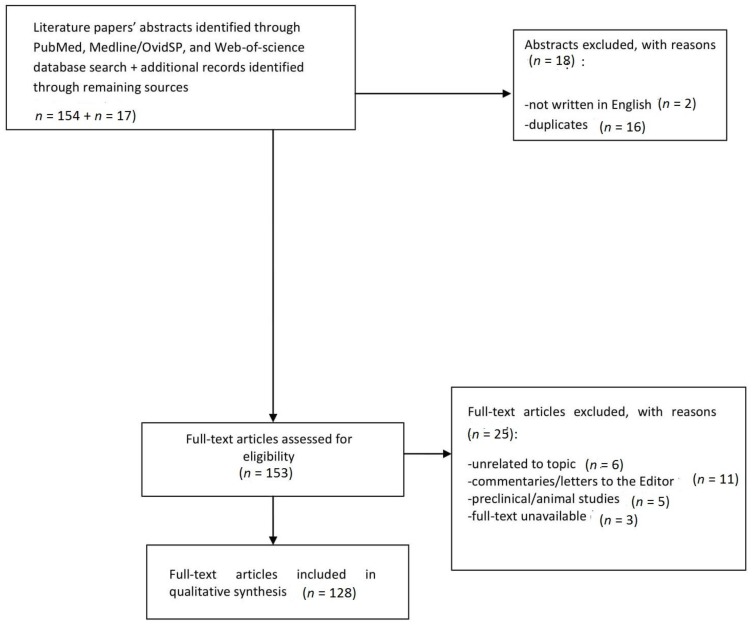
Selection of retrieved studies.

From an initial list of 171 studies, 151 were identified as relevant and appropriate in terms of quality according to PRISMA checklists. Following this, duplicates, papers lacking an English abstract, letters to the editor, animal studies and papers unrelated to the topic were excluded, and 128 papers were finally considered for the current study. A flow diagram (Figure 1) describes the reasons for study inclusion/exclusion at each stage, is here provided.

## 3. Results

### 3.1. Gabapentinoids

Recently, the gabapentinoids, pregabalin and gabapentin, have increasingly been reported to be abused at the EU-wide level, in parallel with increasing levels of prescriptions, related fatalities and a growing black market [16,17,18,19]. Gabapentinoids are anticonvulsants but are also prescribed for a range of clinical conditions in neurology, psychiatry and rheumatology, whilst being used off-label for the treatment of benzodiazepine and alcohol dependence. Their effects are the result of calcium channel binding, resulting in decreased central excitability levels. Compared to gabapentin, pregabalin’s binding affinity and potency are six times higher; pregabalin’s more significant misuse potential may also be due to its more rapid absorption, faster onset of action, much faster attainment of maximum plasma concentration and higher bioavailability (>90%, irrespective of the dosage). Furthermore, gabapentinoids are thought to possess GABA-mimetic properties, whilst possibly having direct/indirect effects on the dopaminergic ‘reward’ system [7]. Gabapentionoid web enthusiasts report the ingestion of this compound alone or in combination with other drugs (e.g., cannabis, alcohol, opioids and other prescribed drugs), at a dosage range of 1000–4800 mg for gabapentin [20], and 750–12,000 mg for pregabalin [7]. Typical psychoactive effects include a sense of well-being/relaxation, euphoria, and even hallucinations [1]. In 2005, the Drug Enforcement Administration (DEA) placed pregabalin into Schedule V of the Controlled Substances Act (CSA) because of its potential for abuse [21] and a similar scheduling approach has recently been approved in the UK. Chiappini and Schifano [7] recently assessed the EMA EV database of pregabalin and gabapentin misuse-related Adverse Drug Reactions (ADRs) over the last decade. According to the Proportional Reporting Ratio (PRR) computation, abuse/dependence issues were more frequently reported for pregabalin compared with gabapentin, hence confirming its higher addictive liability levels [7,22]. Furthermore, Emergency Department presentations involving intentional drug overdoses recorded by the National Self-Harm Registry (Ireland; 2007–2015), showed that gabapentinoids have been increasingly identified over time, with high dosages and polydrug abuse being reported [23]. Indeed, gabapentinoid fatalities are typically observed when these molecules are associated with other psychoactive drugs, especially opioids and other sedatives whose effects are potentiated by gabapentinoids [24,25].

### 3.2. Antidepressants

Consistent with a worldwide rise in antidepressant consumption [26,27], bupropion and venlafaxine have anecdotally emerged as increasingly being abused [1,28,29]. In examining a range of online communities and specialized web services, several antidepressant misusers’ experiences may be identified [20]. These reports emphasise both bupropion’s stimulant effects and venlafaxine’s dissociative properties. Indeed, bupropion described as being ingested in very large quantities (up to 4050 mg/day, roughly 14 times higher than the maximal therapeutic dosage) in order to achieve an ‘amphetamine-like high’ [30]. In most abuse cases, its recreational use is associated with oral or nasal administration, but intravenous use has also been reported [28,30,31,32,33,34]. Bupropion pharmacology relies on its action both as a selective inhibitor of catecholamines (noradrenaline and DA) reuptake [35,36], and as a non-competitive antagonist of nicotinic acetylcholine receptors, hence being prescribed as well as an aid in smoking cessation [36]. Bupropion is known to be a cathinone derivative, that is, a beta-ketone amphetamine analogue with dopaminergic and noradrenergic effects, which may explain its misuse potential [37,38]. This is a reason for concern since bupropion is also used ‘off-label’ in a range of conditions, including attention-deficit/hyperactivity disorder, chronic fatigue, sexual dysfunction, and obesity. The adverse effects of bupropion misuse range from nasal pain to irritability, agitation, cardiac toxicity, hallucinations and seizures [39,40]. A retrospective review [41] on bupropion cases of intentional abuse reported to the US National Poison Data System highlighted an increase of 75% from 2000 to 2012, with the typical effects reported including tachycardia, seizures, agitation/irritability, hallucinations/delusions, and tremor; similar data were identified by the Toxicology Data Network of the US National Institute of Health (Toxnet) [42]. Typical bupropion abusers may present with a history of drug addiction [38,43,44] and/or are inmates, with bupropion having been removed from some US prison formularies [45,46,47]. Conversely, venlafaxine is a selective serotonin-norepinephrine reuptake inhibitor (SNRI) antidepressant, indicated [48] for the treatment of major depressive episodes, generalised anxiety disorder and social phobia, with off-label use including obsessive-compulsive disorder and chronic pain syndromes. Its reuptake effects are dose-dependent, with action progressively including serotonin (5-HT), norepinefrine (NE) and dopamine (DA). Venlafaxine’s main active metabolite, desvenlafaxine, is highly inhibitive of NE transporter activities, further increasing the rate of DA turnover in the prefrontal cortex [49]. Both venlafaxine and its metabolite are not associated with monoamine oxidase inhibitory activity, which is responsible for the degradation of DA. Hence, venlafaxine abuse may be associated with DA increase in the prefrontal cortex [50], high affinity for D2 receptors adaptive changes in D3 receptors following its chronic administration and, finally, with the desensitisation of both 5-HT1A and beta-adrenergic receptors [51]. Dependence and withdrawal symptoms associated with both SSRIs and SNRIs have already been described, specifically with abrupt discontinuation of venlafaxine (including Extended Release (XR) formulation) after long-term use [9,52,53,54]. Symptoms range from mild to severe and include nausea, depression, suicidal thoughts, disorientation, stomach cramps, panic attacks, sexual dysfunction, headaches and occasional psychotic symptoms [55,56,57,58,59]; a newborn discontinuation syndrome has been described as well, at times associated with encephalopathy or paroxysmal episodes [60]. The management of venlafaxine withdrawal includes the use of other antidepressants (Ads) or venlafaxine tapering doses [61,62]. Furthermore, venlafaxine/‘baby ecstasy’ abuse has been reported, typically being the result of the intake of very large doses [63,64,65]. Consistent with this, studies have assessed drug and pharmaceutical consumption in England through wastewater analysis and comparing it to NHS prescription statistics. Discrepancies have been observed in the case of venlafaxine, suggesting sales of non-prescribed venlafaxine, which are, therefore, not included within NHS data [66]. Furthermore, in a retrospective review of the records of the New Zealand National Poisons Centre over the period 2003-2012, rapidly increasing levels of enquiries were identified for a range of prescription medicines, including venlafaxine [67]. According to the EMA EV database from the last decade [68], the misuse-/abuse-/dependence- and withdrawal-related ADRs reported respectively for bupropion and venlafaxine show that bupropion may possess a higher recreational value due to its dopaminergic and stimulant-like activity, whilst the occurrence of a venlafaxine-withdrawal syndrome may be a significant issue for venlafaxine-treated patients; these data were confirmed by analysis of the UK-based Yellow Card Scheme [68].

### 3.3. Antipsychotics

Consistent with their increased prescription and availability [69], second-generation antipsychotic (SGA) (e.g., quetiapine and olanzapine) abuse has recently been reported [1,70,71,72]. Quetiapine appears to be the most documented SGA being abused; it is commonly administered in the 400–800 mg/day range for the treatment of schizophrenia; bipolar disorder; and as an add-on in major depression and anxiety [73,74,75,76]. Quetiapine is anecdotally known as ‘Susie Q’; ‘Quell’; and ‘baby heroin’ [75,76,77,78,79], with ‘Q ball’ and ‘Maq ball’, respectively, being combinations with cocaine, and marijuana. Crushed quetiapine tablets can be self-administered through nasal insufflation [79,80,81], although both oral [81,82,83,84], and intravenous [85,86,87] routes of administration have been reported. Consistent with these anecdotal clinical observations, post-marketing surveillance reports indicate an increase in quetiapine availability on the black-market [75,79,88,89,90]. Furthermore, quetiapine, either on its own or in combination with heroin and/or alcohol [91], is consistently associated with high rates of ambulance attendances, indicating greater community-level harm relative to other atypical antipsychotics [92]. Indeed, between 2005 and 2011, quetiapine-related Emergency Department visits increased in the USA by 90%, from 35,581 to 67,497 attendances [93]. A recent US National Poison Data retrospective analysis identified all cases of single-substance SGA exposures coded as ‘intentional abuse’ [94] during a 10-year period (2003–2013), quetiapine being the most represented molecule, followed by risperidone and olanzapine. Prison inmates and opioid addicts seem to represent the most at-risk populations [24,75,76,95,96,97]. Quetiapine psychotropic effects [86,87] are associated with both increased levels of DA in the nucleus accumbens (NAc) area [89,98,99,100] and D2 receptor blockage. As some pharmacodynamic mechanisms are shared by other non-misused SGAs [101,102,103,104], other factors [105,106] or pharmacological effects explaining the molecule misuse potential may include norquetiapine-related norepinephrine reuptake blockade [75], 5-HT7 antagonist properties and sigma receptor activation [107,108]. Quetiapine pharmacokinetics, mediated by the cytochrome CYP3A4, may play a part, as well, in facilitating its misuse [109]. Its XR formulation may be less frequently abused, due to the delayed (by approximately 3 h) and blunted (by approximately 67%) serum peak [88]; the tablet coating may also make snorting of the crushed tablets quite problematic [89].

Another SGA, olanzapine, is normally prescribed at a dosage of 5–20 mg oral daily in order to treat schizophrenia, bipolar disorder and resistant depression. Whilst being widely prescribed, it has been anecdotally reported, at dosages up to 50 mg, as the ‘ideal trip terminator/modulator’ after a psychedelic drug binge [110]. According to discussion forums/specialised websites [111], olanzapine is also being used to treat unwanted ‘comedown’ symptoms from drug/alcohol intake [112,113]. Consistent with this, clients on methadone maintenance treatment attending the National Drug Treatment Centre (NDTC) in Dublin reported levels of non-medical use of olanzapine, with dosages of up to 100 mg/day, in order to manage anxiety and improve sleep, and in a minority of cases, to ‘get stoned’ [114]. Olanzapine activity involves GABA-A receptors [115], hence the associated sedation, the rewarding glutamatergic stimulation of the ventral tegmental area DAergic neurons [116], the 5HT2C and histamine/H1 antagonist properties and the potent inhibiting action on the muscarinic M1 receptors [115,116]. In comparing quetiapine with olanzapine through the UK Prescription Cost Analysis and the Drug Analysis Profiles of the Yellow Card Scheme, quetiapine was shown to be slightly less frequently prescribed but associated with a smaller total number of general reports, and hence, a comparatively higher number of abuse/dependence/withdrawal ADR reports [117,118]. In line with this, the OPPIDUM French addictovigilance network highlighted the emerging misuse of prescription molecules, and this included quetiapine as well [119]. Information from the previous 10 years from the EMA EV database relating to quetiapine and olanzapine misuse/abuse/dependence/withdrawal-related ADR reports [8] shows a higher misuse risk for quetiapine in comparison with olanzapine for the selected ADR reports. Indeed, quetiapine XR formulation was represented in only a smalll proportion of misuse cases, with both nasal and parenteral administration having been identified. Of particular interest was, in comparison with olanzapine, a higher risk of discontinuation/withdrawal syndrome following the abrupt cessation of quetiapine [75,113,120]. Finally, consistent with previous data [75,82,83,84,85,90,121,122,123], the quetiapine- and olanzapine-related fatalities reported on the EMA EV database were typically the result of a polydrug intake, which included opiates/opioids, antidepressants, and over-the-counter drugs [124,125].

### 3.4. Image-And Performance-Enhancing Drugs (IPEDs)

Over the last few decades, a range of prescribed and non-prescribed enhancement drugs have increasingly been self-administered [72] in order to improve the ageing process, and sexual performances, and to reduce hair loss, fatigue and other physiological conditions which are, at times, considered pathological in a society that strongly emphasises the importance of physical appearance [126]. Prescribed image- and performance-enhancing drugs (IPEDs) include anabolic-androgenic steroids (AAS), human growth hormone (hGH), steroid hormones (e.g., androstenedione), insulin, erythropoietin, diuretics, but also, β-2 agonists (e.g., clenbuterol and salbutamol) [127]. Their misuse is typically carried out within a polypharmacy context [128] with alcohol, cannabis/cannabinoids, cocaine, amphetamines/methamphetamines being ingested as ancillary drugs. Moreover, the recent reporting of IPED injecting practices are a reason for concern [129]; these mostly involve anabolic androgenic steroids, non-steroidal anabolic hormones (e.g., hGH and insulin), tanning peptides, cosmetic injectables such as botox and dermal fillers, etc. [130,131,132]. Among non-steroidal anabolic hormones, insulin seems to be misused for performance-enhancement purposes through several administration routes (intravenous, intramuscular and subcutaneous); indeed, insulin may help in achieving a decrease in fat deposition, an increase in muscle mass and positive mood changes, although serious hypoglycaemic episodes and other medical sequelae can occur as well [133,134]. Within the IPED group, anti-asthmatic beta-2 agonists have recently emerged as having potential for misuse, e.g., salbutamol for its performance-enhancing effects and clenbuterol for its hypertrophic and lipolytic effects. They are both included in the list of prohibited substances released by the World Anti-Doping Agency (WADA) [135], with salbutamol being allowed only as a component in the treatment regimen for athletes with asthma. Clenbuterol, even if different from anabolic steroids, has been also prohibited as an anabolic agent since 2006. In parallel with this, the Food and Drug Administration (FDA) banned the use of clenbuterol in food animals in 1991 and the European Union (EU) followed suit in 1996 [136]. Beta-2 agonists are synthetic molecules with sympathomimetic activity, prescribed as bronchodilators for the treatment of asthma. Clenbuterol is licensed for human use only in a few countries (Austria, Germany, Italy, Spain and Mexico), but not in the UK or the USA [137]. Clenbuterol, as a ‘size zero pill’, is popular and widely available on the web, being considered an ergo/thermogenic drug and hence, an anabolic burner [138], similar to caffeine, ephedrine, and thyroid hormones. Clenbuterol-associated lypolisis can occur via both β-2 adrenergic agonism and its specific action on the adipocytes’ β-3 adrenergic receptors, which further facilitates lipolysis and weight loss [139,140,141]. While anti-asthmatic clenbuterol dosage ranges between 20 and 40mcg daily, the typical ‘fat burning’ dose is in the 120–160 mcg daily range; dosage starts at 40 mcg daily, gradually increases, and then remains at the highest dosage for a duration of 2–4 weeks [142]. In parallel with this, recent years have seen an increase in clenbuterol exposure reported to poison control centres [143], with the molecule being used either as a dietary supplement [144] or as an adulterant in illicit drugs, such as cocaine [145]. Its adverse effects are dose-dependent and may include dysrhythmias and myocardial injury, headache, abdominal pain, nausea, and rhabdomyolysis [136,146,147,148]. Reports relating to salbutamol misuse have been less frequently mentioned [149,150,151]. Similar to clenbuterol, salbutamol’s adverse effects are dose-related and may include tremor, restlessness, anxiety/agitation, tachycardia, atrial fibrillation, and myocardial ischaemia, especially in cases of overdosage, chronic use, or intravenous injection [152]. With respect to salbutamol, clenbuterol’s higher levels of abuse potential could be associated with its pharmacological characteristics [143], such as its prolonged elimination half-life (35 h) and its higher lipophilicity, which can be associated with a fast transition through the blood–brain barrier. Consistent with this, salbutamol has been described as significantly less potent on a reinforcement schedule than clenbuterol [149,152,153,154]. Clenbuterol abuse-related fatalities have consistently been reported in the literature [136,140,147,155], whilst salbutamol is considered safer [156]. In this regard, Milano et al. [10] studied the 2006–2016 EMA clenbuterol- and salbutamol-related, misuse/abuse/dependence/withdrawal/overdose/off-label spontaneous reports. They found that clenbuterol, in comparison with salbutamol, had higher levels of misuse/abuse. These clenbuterol-related data were most typically from males and were associated with the intake of steroids [10], hence confirming previous reports [157,158].

### 3.5. Over-The-Counter (OTC) Medicines—Loperamide

Currently, over-the-counter (OTC) abuse (‘pharming’) is an internationally recognised problem, and the recent emergence of new forms, including online, of medicine supply, is alarming clinicians and health authorities nationwide. The EU introduced a strong legal framework for the licensing, manufacturing and distribution of medicines [159], but no measures have been taken so far for the distribution of OTC drugs, and it is hence, difficult to quantify their actual misuse and abuse [159,160,161,162,163,164,165,166,167,168]. Over previous years, the OTC antidiarrhoeal medicine loperamide has increasingly been reported as being diverted and used to achieve recreational effects [159,160,161,162]. Loperamide acts as a potent mu-opioid receptor agonist, albeit with predominantly peripheral activity on the myenteric plexus, hence primarily increasing the intestinal transit time by decreasing propulsive activity. Secondary peripheral effects are seen at κ-opioid and δ-opioid receptors as well [169,170]. Loperamide was initially placed by the US FDA in Schedule V of the Controlled Substance Act but then, after having assessed its safety profile with the conclusion of low levels of physical dependence risk, in 1988, it was made available for OTC use. In the 2–16 mg daily dosage, loperamide is considered safe and devoid of misuse abuse potential because of its rapid metabolism and poor blood–brain barrier (BBB) penetration. In doses of 50–300 mg, however, loperamide ingestion has been associated with euphoria, central nervous system depression [171,172,173,174] and even death [175]. Its diversion potential may be associated with its use as a relief from opioid withdrawal [176]. Anecdotally described as the ‘poor’s’ methadone’ [177], detailed loperamide dosage titration regimens are being reported online [20]. Related misuse case series [178] have reported both extremely high daily intakes (up to 1200 mg), and associated cardiotoxicity issues, such as QTc prolongation and torsades de pointes, QRS prolongation, ventricular dysrhythmias [179,180,181,182], syncope, and cardiac arrest [12,179,183,184]. The cardiotoxicity mechanism of loperamide is not clearly understood, although it may be due to potent inhibition of cardiac ion channels which is, in turn, associated with delayed repolarisation and QT prolongation [185,186,187]. Consequently, the FDA [175] has recently warned clinicians and users about the combination of loperamide with other drugs or herbal products that are known to prolong the QT interval, including Class 1A (e.g., quinidine, procainamide) or Class III drugs (e.g., amiodarone, sotalol) antiarrhythmics, antipsychotics (e.g., chlorpromazine, haloperidol, thioridazine, ziprasidone), antibiotics (e.g., moxifloxacin), and methadone. Loperamide ingestion has also been reported in association with P-glycoprotein (P-gp) substrates (e.g., quetiapine, cetirizine, oxycodone) or inhibitors (e.g., fluoxetine, citalopram, sertraline, omeprazole, quinine, quinidine, propranolol, ritonavir). These associations are associated with an increase in the low bioavailability of loperamide, normally being <2%; plasmatic concentration; levels of euphoric effects; the capacity of possible contrasting opioid withdrawal symptoms [186,187,188,189]; and toxicity effects [175]. Concurrent use of loperamide with CYP3A4 inhibitors (e.g., itraconazole, grapefruit juice, omeprazole, tonic water and cimetidine) or CYP2C8 inhibitors (e.g., gemfibrozil) can increase its plasma levels as well, with recurrent ventricular tachycardia having been reported in a patient who was taking large recreational doses of both loperamide and the CYP3A4 inhibitor, famotidine [190]. Treatment of loperamide intoxication involves the use of naloxone, which may not be able to directly reverse loperamide cardiotoxic effects [191,192].

## 4. Discussion

The ever-increasing number of NPS emerging worldwide and the parallel changes in drug scenarios represent a challenge for psychiatric, public health and drug-control policies [193]. In line with this, the current systematic review has focused on a different range of prescribed medications which are indeed being used as NPS [1]. Within both online drug forum communities and social networks, there are some educated/informed users (the ‘psychonauts’) [194] who typically ‘test’ a range of psychotropics, including prescribed drugs, to achieve specific mindsets and eventually, share this information with peers [193]. However, in parallel with recently increased levels of access to the web, a large number of vulnerable subjects, including both children/adolescents and psychiatric patients, have been exposed to a range of ‘pro-drug’ information, and this is a reason for concern [193]. Although a number of online ‘rogue’ pharmacies have been shut down, this typically prompts the sellers to move to servers in overseas countries, leading to a growing black market [195].

It is intriguing that, for the range of prescription molecules discussed, including the fairly recently introduced gabapentinoids, pre-marketing processes were not been able to appropriately identify their abuse/misuse potential. However, similar to what happened with benzodiazepines and *z*-hypnotics, this potential has finally emerged over time. Present data seem to suggest that abuse liability-focused, pre-marketing laboratory testing may need to consider interaction studies with alcohol and/or other drugs [194,196]. Furthermore, post-marketing surveillance for substance abuse [197] should routinely be carried out to assess the abuse potential of newly released drugs, especially those with activity on the central nervous system (CNS) [198]. Indeed, lack of information on the abuse/misuse potential of a new medicine’s interaction with the CNS does not mean that a specific medicine does not actually produce these effects. Furthermore, in order to look at how medicines are actually used in real life, modern pharmacovigilance should identify a range of technical tools and approaches to go beyond spontaneous reporting systems. Physicians should be vigilant when prescribing drugs with an abuse/misuse/diversion potential and carefully evaluate the possibility for some clients (inmates; people with a personal history of misuse or abuse) to be more vulnerable to these misuse activities. Finally, while a continuum of related professional training is needed, it may be important to consider a strategy to increase clients’ access to treatment services, possibly through enhanced links between community pharmacists, who are the first professionals to identify a repeat supply issue, and prescribers/clinicians [198].
